# Effectiveness and tolerability of dolutegravir and abacavir/lamivudine administered as two separate pills compared to their equivalent single‐tablet regimen in a multicentre cohort in Spain

**DOI:** 10.1002/jia2.25758

**Published:** 2021-07-22

**Authors:** Inés Suárez‐García, Belén Alejos, Marta Ruiz‐Algueró, Cristina García Yubero, Cristina Moreno, Enrique Bernal, Laura Pérez‐Is, Zuriñe Zubero, Miguel Alberto de Zárraga Fernández, Gloria Samperiz Abad, Inma Jarrín, the Cohort of the Spanish HIV/AIDS Research Network (CoRIS)

**Affiliations:** ^1^ Infectious Diseases Group Department of Internal Medicine Hospital Universitario Infanta Sofía (FIIB HUIS HHEN) Madrid Spain; ^2^ Universidad Europea Madrid Spain; ^3^ National Epidemiology Centre Instituto de Salud Carlos III Madrid Spain; ^4^ Department of Hospital Pharmacy Hospital Universitario Infanta Sofía (FIIB HUIS HHEN) Madrid Spain; ^5^ Infectious Diseases Section Hospital General Universitario Reina Sofía Murcia Spain; ^6^ FINBA/ISPA Hospital Universitario Central de Asturias Avilés Spain; ^7^ Department of Infectious Diseases. Hospital Universitario Basurto Bizkaia Spain; ^8^ Department of Internal Medicine Hospital San Agustín Avilés Spain; ^9^ Infectious Diseases Unit Hospital Universitario Miguel Servet Zaragoza Spain

**Keywords:** generic drugs, single‐tablet regimens, antiretroviral therapy, HIV infections, cohort studies

## Abstract

**Introduction:**

We aimed to assess the effectiveness and tolerability of dolutegravir (DTG), abacavir (ABC) and lamivudine (3TC) administered as branded STR (DTG/ABC/3TC) or as two separate pills (DTG and either branded ABC/3TC [DTG+(ABC/3TC)b] or generic ABC/3TC [DTG+(ABC/3TC)g]).

**Methods:**

We included individuals from the multicentre cohort of the Spanish HIV/AIDS Research Network (CoRIS) who received DTG/ABC/3TC, DTG+(ABC/3TC)b or DTG+(ABC/3TC)g during 2015 to 2018. We used multivariable logistic regression to compare the proportion of antiretroviral‐naïve individuals who achieved viral suppression (VS) (viral load ≤50 copies/mL) at 24 weeks of initiating with DTG+(ABC/3TC)b or DTG+(ABC/3TC)g versus DTG/ABC/3TC. We also calculated the proportion of virologically suppressed individuals who maintained VS at 24 weeks after switching from DTG/ABC/3TC to DTG+(ABC/3TC)g.

**Results:**

During the study period, 829, 68 and 47 treatment‐naïve individuals started treatment with DTG/ABC/3TC, DTG+(ABC/3TC)b or DTG+(ABC/3TC)g respectively. The proportions of individuals who changed their regimens due to side effects during the first 24 weeks were 3.7%, 4.4% and 6.4% respectively (*p* = 0.646). We did not find significant differences in VS at 24 weeks among individuals starting with DTG+(ABC/3TC)b or DTG+(ABC/3TC)g compared to those initiating with DTG/ABC/3TC. Among 177 virologically suppressed individuals who switched from DTG/ABC/3TC to DTG+(ABC/3TC)g, 170 (96.0%) maintained VS at 24 weeks.

**Conclusions:**

In naïve individuals, the effectiveness and tolerability at 24 weeks of DTG plus ABC/3TC administered as two separate pills, either as branded or generic ABC/3TC, was similar to the STR DTG/ABC/3TC. Switching the STR DTG/ABC/3TC to its separate components DTG+(ABC/3TC)g in virologically suppressed individuals did not seem to impair its effectiveness.

## 1 INTRODUCTION

Highly active antiretroviral therapy (ART) has substantially increased the life expectancy of people living with HIV [1, 2]. People living with HIV (PLHIV) have a life expectancy that can reach that of the HIV‐negative population, and ART needs to be maintained for life. Due to the increasing prices of antiretroviral drugs (ARVs) [3, 4], the total healthcare costs for PLHIV are mainly determined by the cost of ART [4, 5].

Since the 1990s, generic ARVs have become available and have shown to be safe and effective for the treatment of HIV infection [6, 7]; these can substantially lower the costs of ART [8, 9]. However, most of the ARV regimens that are administered as fixed‐dose, single‐tablet regimens (STRs) are not available as generics, as usually at least one of their components (such as dolutegravir (DTG) or rilpivirine (RPV)) is still covered under patent protection. Therefore, changing branded STRs for their equivalent generic components often entails administering them as at least two separate pills (these changes are known as “de‐simplifying STRs” or “breaking the combos”). These changes are controversial, and several studies have shown that, while most HIV care clinicians agree on using generic ARVs to decrease healthcare costs, many of them do not agree to de‐simplify STRs, mainly because of concerns of potential decreased treatment adherence [10, 11, 12].

Several studies have contributed to the belief that STRs are more effective than multiple‐tablet regimens for the treatment of HIV infection, as they have shown better adherence [13, 14] and a higher probability of virological suppression than multiple‐tablet regimens [15]. However, most of these studies did not compare STRs with their equivalent separate components; the very few published studies that performed this comparison did not find any significant differences in effectiveness between the two treatment strategies [16, 17, 18, 19, 20]. However, most of these studies have analysed STRs that are no longer recommended as first‐line therapy in high‐income countries [16, 17, 18, 19]. To our knowledge, only two single‐centre studies have evaluated the de‐simplification of the STR dolutegravir/abacavir (ABC)/lamivudine (3TC), with high rates of virological effectiveness in a limited number of patients [20, 21], but data on the comparative effectiveness of DTG/ABC/3TC versus its separate components (DTG+ABC/3TC) in treatment‐naïve patients are lacking.

The aims of our study were (i) to assess the effectiveness and tolerability of dolutegravir, abacavir and lamivudine administered as two separate pills (DTG and either branded or generic ABC/3TC) compared to administering the same regimen as branded single‐tablet regimen (STR) (DTG/ABC/3TC, Triumeq®) in antiretroviral‐naïve individuals, (ii) to assess effectiveness and tolerability of DTG+generic ABC/3TC among virologically suppressed treatment‐experienced individuals who switched from DTG/ABC/3TC to DTG+generic ABC/3TC, and (iii) to assess the cost savings of using DTG+generic ABC/3TC instead of DTG/ABC/3TC for the treatment of HIV infection in the cohort of the Spanish HIV/AIDS Research Network (CoRIS).

## 2 METHODS

### 2.1 Study design

CoRIS is an open, multicentre and prospective cohort of HIV‐positive adults, naïve to antiretroviral treatment at study entry, seen for the first time from 1 January 2004 in any of the 46 centres from 13 of 17 Autonomous Regions in Spain, and followed up until 30 November 2019, the administrative censoring date for these analyses.

Briefly, CoRIS collects a minimum dataset as provided for in the cohort protocol which includes baseline and follow‐up socio‐demographic, immunological and clinical data including ART with its start and stop dates, as well as reasons for drug discontinuation [22].

Data are subjected to internal quality control. Individuals are followed periodically in accordance with routine clinical practice.

### 2.2 Study population

To assess the first objective, we included antiretroviral‐naïve individuals who started ART with DTG, ABC and 3TC, either administered as STR (DTG/ABC/3TC, Triumeq®) or as two separate pills (DTG and either branded ABC/3TC [DTG+(ABC/3TC)b] or generic ABC/3TC [DTG+(ABC/3TC)g]) between 1 January 2015 and 31 December 2018.

For the second objective, we included virologically suppressed individuals (defined as viral load (VL) ≤50 copies/mL) who switched from DTG/ABC/3TC to DTG+(ABC/3TC)g between 1 January 2015 and 31 December 2018.

For the third objective, we included antiretroviral‐naïve individuals initiating ART with DTG/ABC/3TC or DTG+(ABC/3TC)g during the years 2017 and 2018, when generic ABC/3TC was available in Spain.

For all the analyses, we excluded individuals (i) under 18 years of age, (ii) with no follow‐up after ART initiation, (iii) from six centres that did not report information on the use of generic antiretroviral drugs (ARVs), (iv) with VL ≤ 50 copies/mL at ART initiation as it was considered to be a probable error in the data, (v) who were receiving ART in the context of a clinical trial and (vi) with no information on VL at 24 weeks (±12 weeks) from ART initiation or switch, as appropriate.

### 2.3 Statistical analysis

Descriptive analyses were carried out using frequency tables for categorical variables and median and interquartile range (IQR) for continuous variables. Differences in socio‐demographic and clinical characteristics according to initial regimen were assessed with the nonparametric Mann–Whitney or Kruskal–Wallis test, as appropriate, for continuous variables and the chi‐squared test for independence for categorical variables.

To assess treatment effectiveness, for both antiretroviral‐naïve and treatment‐experienced individuals, we calculated the proportion of individuals who achieved or maintained viral suppression (VS), defined as VL ≤ 50 copies/mL, at 24 (±12) weeks after initiation or switch of ART respectively.

For treatment‐naïve individuals, multivariable logistic regression models were used to estimate the association between initial regimen and VS, adjusting for the following potential confounders: sex (male, female), age at ART initiation (<30, 30 to 49, ≥50 years), transmission category (men who have sex with men (MSM), heterosexual, other/unknown), educational level (no education or compulsory education, upper secondary or university education, other/unknown), country of origin (Spain, foreign‐born, unknown), CD4 count (<200, 200 to 500, >500 cells/μL, unknown) and HIV VL (<100,000, ≥100,000 copies/mL, unknown) within six months previous to ART initiation, and AIDS diagnosis at the initiation of ART (no, yes). Crude and adjusted odds ratios (ORs) were calculated. To adjust for clustering of individuals within centres, robust methods were used to estimate standard errors and, thus, to calculate 95% CI and *p* values. Wald tests were used to derive *p* values.

To assess treatment tolerability, we calculated the proportion of individuals who changed their treatment during the first 24 weeks after ART initiation (for treatment‐naïve individuals) or switch (for virologically suppressed pre‐treated individuals) as well as the reason for the change and the substitution regimen. Reasons for the change were classified as treatment failure, side effects, STR introduction, STR de‐simplification, other and unknown.

We performed both an intention‐to‐treat analysis (ITT), ignoring treatment changes (once a participant started a regimen, he/she was assumed to remain on it), and an as‐treated analysis (AT) excluding individuals who changed their treatment before 24 weeks. For the AT analysis, individuals changing to different presentations of the same treatment components (e.g. those changing from DTG/ABC/3TC to DTG+(ABC/3TC)g) were considered to have changed their treatment and therefore were also excluded from the analysis.

For the cost analysis, we calculated (1) the annual savings during the first year of treatment achieved by prescribing DTG+(ABC/3TC)g instead of DTG/ABC/3TC to the included patients, and (2) what the annual savings would have been for the Spanish National Health System (NHS) during the first year of treatment if all the antiretroviral‐naïve individuals in the CoRIS cohort who started treatment with DTG/ABC/3TC during the years 2017 and 2018 had started with DTG+(ABC/3TC)g. This reduction was calculated in terms of the pharmacological cost of ART, ignoring costs associated with the clinical management and adverse effects, as it has been shown that the cost for the health system of the first year of ART is mainly driven by the price of drugs [5]. For these analyses, we assumed that once a patient had started treatment with DTG/ABC/3TC or DTG+(ABC/3TC)g, he/she would remain on it during the first year of treatment. The annual cost of each regimen per patient was estimated as the best purchase price among all hospitals in the Madrid Autonomous Region as provided by the Subdirección General de Farmacia y Productos Sanitarios del Servicio Madrileño de Salud in 2018 [23].

All statistical analyses were performed using Stata software (version 15.0; Stata Corporation, College Station, TX, USA).

## 3 RESULTS

### 3.1 Effectiveness and tolerability of DTG+ABC/3TC compared to DTG/ABC/3TC in treatment‐naïve individuals

During the study period, 1454 antiretroviral‐naïve individuals aged ≥18 years initiated treatment with DTG, ABC and 3TC. Of those, 510 were excluded from the analysis of the first objective for the following reasons: 35 (2.4%) with no follow‐up after ART initiation, 101 (6.9%) from centres not reporting information on the use of generic drugs, 19 (1.3%) with VL ≤ 50 copies/mL at ART initiation, 86 (5.9%) who were receiving ART in the context of a clinical trial and 269 (18.5%) with no information on VL at 24 weeks from ART initiation.

Finally, 944 antiretroviral‐naïve individuals were included: 829 (87.8%) initiated treatment with DTG/ABC/3TC, 68 (7.2%) with DTG+(ABC/3TC)b, and 47 (5.0%) with DTG+(ABC/3TC)g. Males were 90.7% of the participants and 60.5% were from Spain. The transmission route was homo/bisexual contact in 73.5% of the individuals and heterosexual contact in 19.2%. At treatment initiation, the median age was 36 years (interquartile range [IQR]: 30; 44), median CD4 count was 420 cells/μL (IQR: 273; 596), 6.1% individuals had a history of an AIDS‐defining illness and 38.1% had a VL > 100,000 copies/mL. Socio‐demographic and clinical characteristics at ART initiation according to the initial regimen are shown in Table 1. We did not find any differences between the individuals who initiated each regimen.

**Table 1:** Socio‐demographic and clinical characteristics at ART initiation according to initial regimen in the Spanish CoRIS cohort, 2015 to 2018 (n = 944)

Variable	DTG/ABC/3TC N = 829		DTG+ABC/3TC N = 115			DTG+(ABC/3TC)b N = 68		DTG+(ABC/3TC)g N = 47		
	N	%	N	%	*P*_1_	N	%	N	%	*P*_2_
Sex					0.661					0.763
Male	753	90.8	103	89.6		60	88.2	43	91.5	
Female	76	9.2	12	10.4		8	11.8	4	8.5	
Age (years)					0.205					0.432
<30	223	26.9	34	29.6		19	27.9	15	31.9	
30 to 49	501	60.4	73	63.5		43	63.2	30	63.8	
≥50	105	12.7	8	7.0		6	8.8	2	4.3	
HIV transmission					0.979					0.814
MSM	609	73.5	85	73.9		48	70.6	37	78.7	
Heterosexual	162	19.5	23	20.0		15	22.1	8	17.0	
Other/unknown	58	7.0	3	2.6		5	7.4	2	4.3	
Level of education					0.399					0.488
No/compulsory education	163	19.7	25	21.7		17	25.0	8	17.0	
Upper secondary/university	491	59.2	63	54.8		38	55.9	25	53.2	
Other/unknown	175	21.1	0	0		13	19.1	14	29.8	
Geographical origin					0.627					0.402
Spain	499	60.2	72	62.6		39	57.4	33	70.2	
Migrant	327	39.4	42	36.5		28	41.2	14	29.8	
Unknown	3	0.4	1	0.9		1	1.5	0	0	
CD4 count (cells/µL), median (IQR)	420 (274; 595)		428 (251; 647)		0.878	428 (272; 669)		388 (210; 556)		0.986
Viral load (copies/mL), median (IQR)	57,459 (14,435; 200,000)		79,300 (22,246; 200,000)		0.592	65,600 (22,246; 190,000)		90,400 (26,810; 210,000)		0.777
AIDS					0.616					0.339
No	778	93.8	108	93.9		62	91.2	46	97.9	
Yes	51	6.2	7	6.1		6	8.8	1	2.1	

1 *p*‐value for the comparison between DTG/ABC/3TC and DTG+ABC/3TC. 2 p‐value for the comparison between DTG/ABC/3TC, DTG+(ABC/3TC)b and DTG+(ABC/3TC)g. 3TC, lamivudine; ABC, abacavir; b, branded; DTG, dolutegravir; g, generic; MSM, men who have sex with men.

During the study period, the three different treatment regimens were used in several of the 38 participating centres. DTG/ABC/3TC was used in 36 of the centres, and it was not available in two centres. DTG+(ABC/3TC)b was used in 17 centres: in two centres it was used because DTG/ABC/3TC was not available throughout the whole study period, and in 15 centres it was used before DTG/ABC/3TC was available at their hospital pharmacies. DTG+(ABC/3TC)g was used in eight of the centres, as part of cost containment policies, after 31 January 2017, when (ABC/3TC)g became commercially available in Spain. Virologically suppressed patients who switched from DTG/ABC/3TC to its equivalent two‐drug regimen did so using DTG+(ABC/3TC)g in all cases.

**Figure 1:**
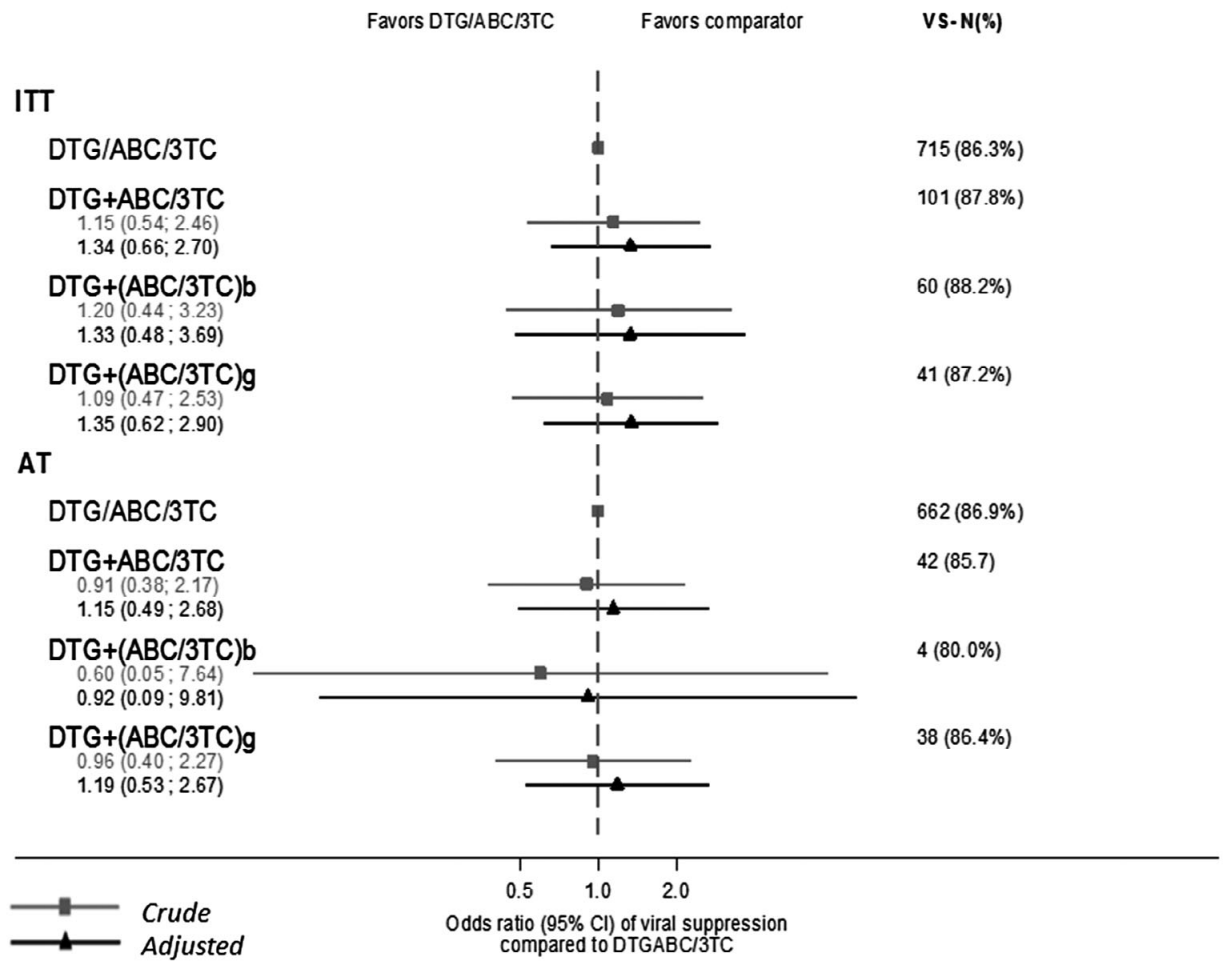
Viral suppression at 24 weeks from ART initiation according to initial regimen in the Spanish CoRIS cohort, 2015 to 2018 (n = 944). 3TC, lamivudine; 95% CI, 95% confidence interval; ABC, abacavir; b, branded; DTG, dolutegravir; g, generic; OR, odds ratio; VS, Viral Suppression

At 24 weeks from ART initiation, 715 (86.3%) individuals initiating DTG/ABC/3TC and 101 (87.8%) initiating DTG+ABC/3TC (88.2% DTG+(ABC/3TC)b and 87.2% DTG+(ABC/3TC)g) achieved VS. In multivariable analysis, we did not find significant differences in VS among individuals who started either DTG+(ABC/3TC)b or DTG+(ABC/3TC)g compared to those who started DTG/ABC/3TC, either as ITT or as AT (Figure 1).

The proportion of treatment changes during the first 24 weeks after ART initiation, as well as the reason for the change and substitution regimen, according to the initial regimen are shown in Table 2. Most individuals (86.8%) starting with DTG+(ABC/3TC)b started treatment when DTG/ABC/3TC was not available in their centres and switched to DTG/ABC/3TC when it became available. The proportions of individuals who changed their regimen due to adverse effects among those starting with DTG/ABC/3TC, DTG+(ABC/3TC)b and DTG+ABC/3TCg were 3.7%, 4.4% and 6.4% respectively (*p* = 0.646).

**Table 2:** Treatment changes during the first 24 weeks after ART initiation, as well as the reason for the change and substitution regimen, according to initial regimen in the Spanish CoRIS cohort, 2015 to 2018 (n = 944)

	DTG/ABC/3TC	DTG+(ABC/3TC)b	DTG+(ABC/3TC)g
	N = 829	N = 68	N = 47
Treatment change – n (%)	67 (8.1)	63 (92.6)	3 (6.4)
Reason for treatment change – n (%)			
Treatment failure	4 (0.5)	0	0
Side effects	31 (3.7)	3 (4.4)	3 (6.4)
STR introduction	0	59 (86.8)	0
STR de‐simplification	11 (1.3)	0	0
Other	18 (2.2)	1 (1.5)	0
Unknown	3 (0.4)	0	0
Substitution regimen – n (%)			
	EVG/COBI/FTC/TAF – 11 (1.3)	DTG/ABC/3TC – 59 (86.8)	EVG/COBI/FTC/TAF – 1 (1.2)
	DTG+(ABC/3TC)g – 11 (1.3)	Other – 5 (7.4)	DTG+FTC/TAF – 1 (1.2)
	DTG+FTC/TDF – 7 (0.8)		DTG+FTC/TDF – 1 (1.2)
	Other – 38 (4.6)		

3TC, lamivudine; ABC, abacavir; b, branded; COBI, cobicistat; DTG, dolutegravir; EVG, elvitegravir; FTC, emtricitabine; g, generic; TAF, tenofovir alafenamide; TDF, tenofovir disoproxil fumarate.

### 3.2 Effectiveness and tolerability of switching DTG/ABC/3TC to DTG+(ABC/3TC)g in virologically suppressed individuals

For the analysis of the second objective, we included 177 virologically suppressed individuals who switched from the STR DTG/ABC/3TC to its separate components DTG+(ABC/3TC)g during the study period. Among those, 170 (96.0%) maintained VS at 24 weeks from switching to DTG+(ABC/3TC)g as ITT analysis, and a similar percentage was found using the AT approach (155 [95.7%] out of 161 individuals). A total of 16 (9.0%) individuals changed their treatment during the first 24 weeks after the switch from DTG/ABC/3TC to DTG+(ABC/3TC)g. Among these individuals, the reasons for change were side effects (5, 2.8%), simplification (5, 2.8%), prevention of side effects (3, 1.7%), costs (1, 0.6%) and other reasons (2, 1.1%).

### 3.3 Cost savings of using DTG+(ABC/3TC)g instead of DTG/ABC/3TC in treatment‐naïve individuals

To calculate the annual savings during the first year of treatment achieved by prescribing DTG+(ABC/3TC)g instead of DTG/ABC/3TC, we included 47 patients who started ART with DTG+(ABC/3TC)g during the years 2017 (n = 12) and 2018 (n = 35). The annual treatment cost per patient of DTG+(ABC/3TC)g and DTG/ABC/3TC during the study period was EUR 5435 and EUR 6450 respectively. The annual treatment cost of DTG+(ABC/3TC)g was 1015 € lower per patient compared to that of DTG/ABC/3TC. Treating those 47 patients with DTG+(ABC/3TC)g instead of DTG/ABC/3TC achieved a cost reduction of EUR 47,705 during their first year of treatment (12,180 in 2017 and 35,525 in 2018).

To calculate the potential cost savings that could be achieved if all the patients in the cohort had started ART with DTG+(ABC/3TC)g instead of DTG/ABC/3TC, we included 410 antiretroviral‐naïve individuals who started ART with DTG/ABC/3TC during the years 2017 (n = 238) and 2018 (n = 172). If these 410 individuals had received DTG+(ABC/3TC)g instead of DTG/ABC/3TC, the cost of ART during their first year of treatment would have been reduced by EUR 416,150 (241,570 in 2017 and 174,580 in 2018).

## 4 DISCUSSION

In this multicentre national cohort, the effectiveness of DTG plus ABC/3TC, administered as two separate pills (either as branded or generic ABC/3TC), did not differ from that of the branded STR DTG/ABC/3TC at 24 weeks. Also, switching the branded STR DTG/ABC/3TC to its separate components DTG plus (ABC/3TC)g in virologically suppressed patients did not seem to impair its effectiveness, as most of the patients remained virologically suppressed at 24 weeks after treatment de‐simplification.

During the study period, two different two‐drug regimens were used. Among treatment‐naïve patients, DTG+(ABC/3TC)g was used in eight centres, as part of cost containment policies, after January 2017, when (ABC/3TC)g became commercially available in Spain. However, with the exception of two centres where DTG/ABC/3TC was not available throughout the study period, DTG+(ABC/3TC)b was used in 15 centres before the STR DTG/ABC/3TC was introduced in these hospitals; therefore, in most of the patients starting this regimen, it was subsequently changed to DTG/ABC/3TC, when it became available in each centre. Virologically suppressed patients who switched from DTG/ABC/3TC to its equivalent two‐drug regimen used DTG+(ABC/3TC)g in all cases.

The tolerability of both two‐drug regimens was good in our cohort. Among treatment‐naïve patients, we found that, excluding those patients who switched from DTG+(ABC/3TC)b to DTG/ABC/3TC when it became available in their centre, treatment changes due to other reasons were low for patients who started either of the two‐drug regimens, with no changes due to virological failure and only 4.4% and 6.4% of patients changing due to adverse effects in the DTG+(ABC/3TC)b and DTG+(ABC/3TC)g groups respectively.

The estimated number of PLHIV in Spain is 146,000 [24], who will need ART throughout their lives. In Spain, where HIV care is provided mainly in the public healthcare system and ART is provided free of charge for all patients, the total cost of ART has been consistently increasing and had an annual cost of EUR 734,367,344 in 2016 [25]. It has been estimated that ART accounts for 73% of the total healthcare costs of PLHIV in the United States [4] and 87% of the healthcare costs in Spain during the first year of treatment [5]. ART is a proven cost‐effective intervention [26], but in a setting of continuously increasing prices and limited resources, new ways to provide affordable and effective ART, such as the use of generic ARVs, are needed.

In the European Union, the use of a generic drug is accepted if it has the same quantity of the active substance, same pharmaceutical presentation, and similar bioavailability to its branded equivalent [27]. However, the de‐simplification of STRs to their separate components is controversial, as it entails a change in the number of pills [27], and there are concerns about worse adherence with multiple‐tablet regimens [28]. Compared to multiple‐tablet regimens, STRs have shown better adherence [13, 14], and lower risk of hospitalizations [13, 29]. In a recent meta‐analysis, STRs showed better adherence, higher probability of VS and lower costs than multiple‐tablet regimens, but there were no differences in immunological response, mortality, adverse effects or tolerability [15].

Based on the aforementioned studies, most clinicians caring for PLHIV are quite confident about prescribing generic ARVs, but they do not agree on de‐simplifying STRs and increasing the daily pill burden of ARVs [10, 11, 12]. However, most of these studies were done in the context of simplification strategies and did not compare STRs with their equivalent separate components, but rather with multiple‐tablet regimens that had different drug combinations, or even different ARV drug classes. There are very few published observational studies comparing STRs with their equivalent separate components. Most of these studies compared the STR efavirenz/tenofovir disoproxil fumarate/emtricitabine (EFV/TDF/FTC) with its separate components, and none of them found any significant differences in the effectiveness of both treatment strategies [17, 18, 19].

To our knowledge, the de‐simplification of the STR DTG/ABC/3TC to DTG+(ABC/3TC)g has only been evaluated by two single‐centre studies in pre‐treated patients: both found this strategy to be safe and effective [20, 21]. Olalla *et al*. found that during the first six months after switching to DTG+(ABC/3TC)g among 93 patients that were receiving DTG/ABC/3TC, no patients had VL above 400 copies/mL (five of the patients had VL between 50 and 400 copies/mL), and only two patients interrupted their treatment due to central nervous system side effects; the adherence measured in the hospital pharmacy was 98% before and after the switch [21]. Krentz *et al*. compared 441 patients who remained on DTG/ABC/3TC to 257 patients who either initiated or switched to DTG+(ABC/3TC)g and found that 3.6% and 1.2% of patients discontinued therapy and 3.4% and 1.2% of patients had any viral load >500 copies/mL respectively [20]. Despite not being able to compare their results with ours (as in their study the time points for assessing these outcomes were not defined, and both pre‐treated patients and a minority of 67 treatment‐naïve patients were not analysed separately), their findings showed high effectiveness and safety of DTG+(ABC/3TC)g. This is, to our knowledge, the first study assessing the effectiveness of DTG+ABC/3TC compared to their equivalent STR among treatment‐naïve patients.

With the limitations of observational studies, our results and the published literature support the effectiveness and tolerability of DTG+(ABC/3TC)g, which can be used in order to decrease healthcare costs for the public healthcare system. Previous studies have suggested the potential savings associated with the use of generic ARVs and the de‐simplification of STRs. A cost‐benefit analysis from the United States estimated a cost reduction of USD 42,500 per patient and a total cost reduction of USD 920,000,000 for the healthcare system if the branded STR Atripla® (including EFV/TDF/FTC) would be switched to its three separate components including generics (generic EFV, TDF and generic FTC) [8]. Also, a French study showed important savings for the healthcare system with the switch to generic‐based ARV regimens, including de‐simplification of EFV/TDF/FTC [9]. Regarding DTG/ABC/3TC, Krentz *et al*. achieved important savings after its voluntary de‐simplification in a Canadian cohort [20].

Some authors have suggested that the cost savings achieved by the de‐simplification of STRs might be lower than expected, as patients switching to multiple‐tablet regimens might experience more side effects and use more healthcare services [19, 30]. However, some bias might have existed in these studies as patients who were known to have de‐simplified their treatment might have had a different assessment and follow‐up by their physicians than those who did not. Our study only estimated the direct costs of the ART and did not assess the cost of use of healthcare services or adverse effects; however, we believe that adverse effects might not have influenced our results as interruptions due to these were infrequent and in similar proportions among the three treatment regimens.

Our study has several strengths. It is a large multicentre cohort with reasonably large sample size, with strict quality control procedures and largely representative of the newly diagnosed PLHIV in the Spanish general population [31]. Our results are limited by the lack of data on adherence to ART, as it is not routinely registered in the cohort; however, we could infer that there were no substantial differences in treatment adherence as we found no significant differences in VS among the three regimens. Also, our findings are limited to a follow‐up period of 24 weeks and we cannot exclude that differences in adherence could become apparent (and therefore have an impact on VS) after a longer follow‐up period.

We need to consider several facts that might limit the generalizability of our findings. Spain is a high‐income country [32] and ART is provided free of charge for the patients in the public healthcare system; therefore, our results might not apply to low‐ or middle‐income countries, or those with different health systems or drug pricing policies. Also, similar to the new HIV diagnoses in the general Spanish population [31], our sample was mainly composed of men and specifically MSM, and the adherence to ART could differ in other settings.

## 5 CONCLUSIONS

In conclusion, our study found no differences in the effectiveness of DTG+ABC/3TC administered as two separate pills compared to the equivalent STR among treatment‐naïve patients, with low rates of discontinuations due to adverse effects. The de‐simplification of DTG/ABC/3TC to DTG+(ABC/3TC)g also seemed to be effective and well‐tolerated among virologically suppressed patients. Given these findings, the de‐simplification of DTG/ABC/3TC for its equivalent separate components including generics among treatment‐naïve and treatment‐experienced patients could be considered as a potential strategy for decreasing costs for the healthcare system.

## Competing interest

ISG has received conference or speaker fees from Viiv, MSD and Gilead. CGY has received conference fees from Gilead and Janssen. IJ has received teaching fees from Viiv and advisory fees from Gilead. All other authors: none to declare.

## Authors’ contributions

All authors were involved in setting up the cohort and contributed to its design. All authors were involved in data collection. ISG and IJ asked the research question presented in this paper and designed the study. BAF performed the statistical analysis with support from IJ. ISG and BAF wrote the first draft of the paper with support from IJ. All authors were involved in the interpretation of the data and commented on interim drafts. All authors have read and approved the final manuscript.

## Supporting information


**Appendix** **S1**. CoRIS participating centres.
